# Mesalamine-Induced Eosinophilic Pneumonia

**DOI:** 10.7759/cureus.94493

**Published:** 2025-10-13

**Authors:** Monish Sharma, Joseph E Sotelo, Steve Schultz

**Affiliations:** 1 Radiology, Texas College of Osteopathic Medicine, Fort Worth, USA; 2 Radiology, University of North Texas Health Science Center, Fort Worth, USA; 3 Radiology, Radiology Associates of North Texas, Fort Worth, USA

**Keywords:** adverse effects of mesalamine, drug-induced pneumonia, eosinophilia, pneumonia, ulcerative colitis

## Abstract

Eosinophilic pneumonia encompasses a group of lung disorders characterized by peripheral blood eosinophilia. While its etiology is often idiopathic or infectious, non-infectious causes, including drug-induced reactions, should also be considered. Common culprits of drug-induced eosinophilic pneumonia include daptomycin, phenytoin, and nitrofurantoin. Mesalamine-induced eosinophilic pneumonia is rare, with only a few cases documented in the literature. We present the case of a 27-year-old male patient who developed unilateral eosinophilic pneumonia following three months of mesalamine therapy for ulcerative colitis. Subsequent discontinuation of mesalamine led to rapid resolution of his symptoms, supporting a diagnosis of mesalamine-induced eosinophilic pneumonia.

## Introduction

Eosinophilic pneumonia is characterized histologically by marked infiltration of eosinophils within the alveolar spaces, bronchial walls, and interstitium. Acute eosinophilic pneumonia develops rapidly, typically within two weeks, and presents with symptoms such as cough, fever, pleuritic chest pain, dyspnea, and night sweats. It is possible for acute eosinophilic pneumonia to progress to respiratory failure. In contrast, chronic eosinophilic pneumonia has a more gradual, subacute onset over several months and is often accompanied by additional symptoms such as weight loss and wheezing. Acute eosinophilic pneumonia is possible at any age; however, it is more common in men between the ages of 20-40. Chronic eosinophilic pneumonia is most common amongst white women with a peak incidence between 30 and 40 years of age. Additionally, it is important to note that asthma is present in 50% of these patients [1]. Although parasitic infections are the most common cause of infectious eosinophilic pneumonia, non-infectious etiologies-such as drug-induced reactions-should also be taken into account during etiological evaluation [1]. Confirmatory testing for eosinophilic pneumonia includes a lung biopsy showing eosinophilic invasion or over 500 x 10 cells/L in bronchoalveolar lavage (BAL) fluid [2]. Differential diagnoses include but are not limited to asthma, fungal infection, interstitial lung disease, and malignancy [1]. Mesalamine, a drug commonly used to treat inflammatory bowel diseases such as ulcerative colitis, has many well-studied side effects. Some of the more common side effects include gastrointestinal symptoms such as nausea, diarrhea, abdominal pain, and respiratory symptoms resembling cold/flu-like symptoms, as well as lung injury [3]. However, through our literature review, we have only found a handful of cases that show mesalamine-induced eosinophilic pneumonia, making this a rare complication that should not be overlooked.

## Case presentation

**Figure 1:**
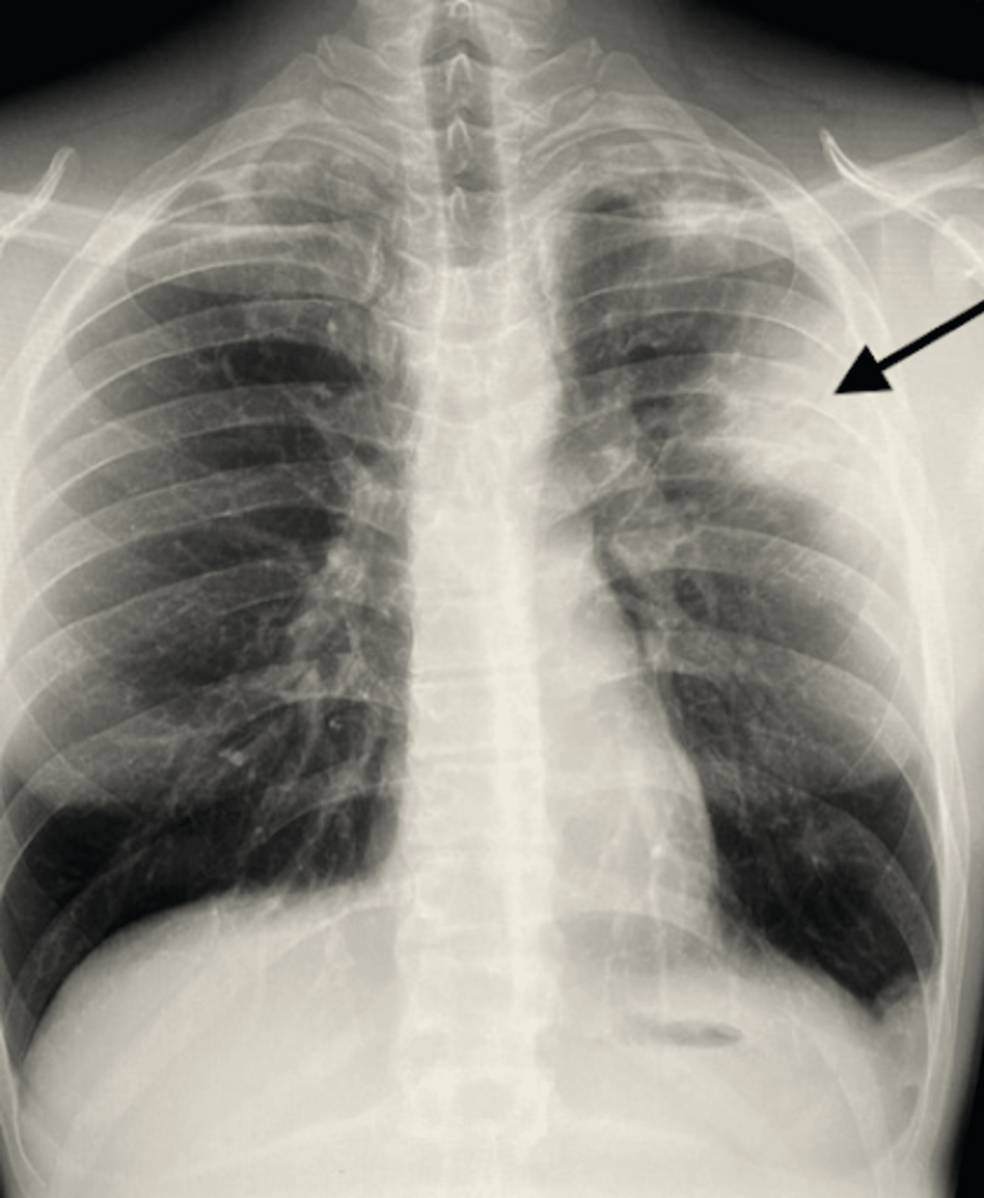
Chest X-ray done on 5/16/2022 showing a unilateral homogenous non-segmental left upper lobe Loeffler-type consolidation pattern (black arrow).

**Figure 2:**
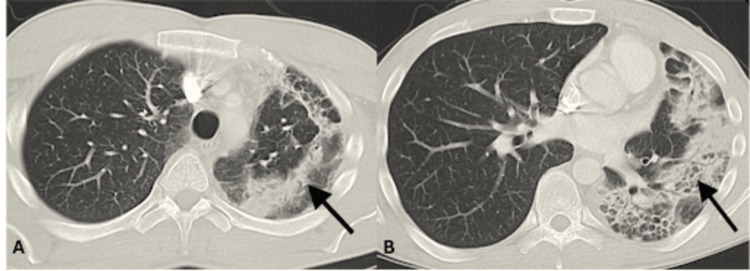
CTA done on 6/10/2022 showing left peripheral parenchymal infiltration (black arrow). CTA: computed tomography angiography

A 27-year-old man presented with a 2-3 week history of cough, fatigue, and chest tightness. He was otherwise healthy with the exception of a diagnosis of ulcerative colitis three months prior, placed on mesalamine treatment with excellent response and resolved GI symptoms. CXR 5/16/22 (Figure 1) showed peripheral left upper lobe consolidation, which was suspicious for inflammatory/infectious processes such as pneumonia. He was not responding to standard antibiotic treatment with worsening of respiratory symptoms. Subsequent computed tomography angiography (CTA) was performed on 6/10/22 (Figure 2), which showed more extensive unilateral Loeffler-type left lung parenchymal infiltration with evidence of cicatrization atelectasis with decreased volume of left hemithorax with mediastinal shift to the left, as seen on original radiography. A subsequent lung specimen was obtained, which showed extensive eosinophilic infiltration. The diagnosis was made as eosinophilic pneumonia complicating his mesalamine treatment. The patient quickly responded to drug discontinuation.

## Discussion

Eosinophilic lung diseases are a heterogeneous group of disorders that can be categorized by meeting one of the following three criteria: (i) peripheral blood eosinophilia accompanied by pulmonary infiltrates on chest X-ray; (ii) a lung biopsy showing a tissue-to-blood eosinophil ratio greater than 100:1; or (iii) an increased percentage of eosinophils in BAL fluid [3]. Eosinophilic pneumonia is characterized histologically by marked infiltration of eosinophils within small airways, such as bronchioles. Parasitic infections are the most common cause of infectious eosinophilic pneumonia; however, non-infectious etiologies, such as drug-induced reactions, should also be taken into account during etiological evaluation [1]. Drug-induced eosinophilic pneumonia is rare, with only 228 cases being reported between 1990 and 2017 [4]. 

Mesalamine is a drug commonly used to treat inflammatory bowel diseases such as ulcerative colitis. Some of the more common side effects of mesalamine include gastrointestinal symptoms such as nausea, diarrhea, abdominal pain, and respiratory symptoms, as well as lung injury [2]. Inflammatory bowel diseases such as ulcerative colitis may cause extra-intestinal manifestations, including pulmonary manifestations. However, this should be differentiated from drug-related lung disease that can occur secondary to the medications used as maintenance therapy for inflammatory bowel disease. Implicated drugs include azathioprine, 6-mercaptopurine, sulfasalazine, mesalamine, and methotrexate. Regarding sulfasalazine and mesalamine specifically, commonly reported lung pathology includes interstitial disease, eosinophilic pleuritis, bronchiolitis obliterans, and eosinophilic pneumonia. Common presentations of these conditions include respiratory symptoms such as dyspnea, cough, chest pain, and radiographic abnormalities. Symptoms often develop within 2-6 months of drug use [5].

The majority of cases of mesalamine-induced lung injury improved with discontinuation of the drug or low-dose corticosteroids. The Japanese consensus statement regarding treatment of drug-induced lung injury proposes methylprednisolone pulse therapy (500-1000 mg per day for three days) followed by 0.5-1.0 mg/kg/day of prednisolone for patients with severe respiratory failure [6]. The patient presented in this case showed rapid improvement in symptoms with drug discontinuation alone.

Furthermore, the typical radiographic appearance of drug-induced eosinophilic pneumonia is that of bilateral nonsegmental homogenous consolidation in the lung periphery, referred to as a reversed pulmonary edema pattern [7]. The opacities are bilateral in at least 50% of cases of eosinophilic pneumonia on chest X-ray, and the proportion of bilateral opacities increases up to more than 95% on high-resolution computed tomography [8]. This case is unique in that there are few reported cases of unilateral lung involvement in patients with drug-induced eosinophilic pneumonia.

## Conclusions

This case highlights a rare but important complication of mesalamine therapy-unilateral eosinophilic pneumonia. In patients presenting with new or worsening respiratory symptoms while undergoing treatment for inflammatory bowel disease, clinicians should maintain a high index of suspicion for drug-induced pulmonary toxicity, especially when symptoms are unresponsive to standard therapies. Early recognition and prompt discontinuation of the offending agent are crucial, as they may lead to rapid clinical improvement and obviate the need for more aggressive interventions. This case contributes to the limited but growing body of literature on mesalamine-induced eosinophilic pneumonia and reinforces the importance of considering medication side effects in the differential diagnosis of atypical pulmonary presentations.
